# In vitro investigation of the effect of laser on platelet-rich fibrins

**DOI:** 10.1186/s12903-026-07974-8

**Published:** 2026-02-24

**Authors:** Utku Böcüoğlu, Esra Ateş Yıldırım, Selma Erdoğan Düzcü, Mustafa Tunalı

**Affiliations:** 1 Research Assistant, Department of Periodontology, Faculty of Dentistry, Bolu Abant İzzet Baysal University, Bolu, Turkey; 2 Assistant Professor, Department of Periodontology, Faculty of Dentistry, Bolu Abant İzzet Baysal University, Bolu, 14300 Turkey; 3 Associate Professor, Department of Medical Pathology, Faculty of Medicine, Bolu Abant İzzet Baysal University, Bolu, Turkey; 4 Professor, Department of Periodontology, Faculty of Dentistry, Çanakkale Onsekiz Mart University, Çanakkale, Turkey

**Keywords:** L-PRF, T-PRF, Platelet-rich fibrin, Laser, Biostimulation

## Abstract

**Objectives:**

This in vitro study aimed to evaluate the effect of laser photobiomodulation on the structural integrity and degradation resistance of two types of platelet-rich fibrin membranes: Leukocyte- and Platelet-Rich Fibrin (L-PRF) and Titanium-Prepared Platelet-Rich Fibrin (T-PRF). Structural changes in the fibrin network were assessed using Scanning Electron Microscopy (SEM) and light microscopy.

**Materials and methods:**

Our study was performed on 15 systemically healthy individuals and four L-PRF and four T-PRF membranes obtained from each individual, totaling 120 samples. L-PRF was prepared first using standard vacuum glass tubes. Two weeks later, new blood samples were collected from the same individuals, and T-PRF membranes were prepared using sterile titanium tubes to enhance biocompatibility. Both membrane types were obtained by centrifugation at 2700 revolutions per minute (rpm) for 12 min. Two of the four membranes were treated with a diode laser device at a wavelength of 980 nanometers (nm) and a power of 0.5 W (W) in continuous mode for 3 min at a distance of 1–2 milimeters (mm). The other two membranes were not lasered. One of the laser treated L-PRF and T-PRF membranes was cut in half and stored under appropriate conditions for histological examination and SEM analysis. The other membrane was separated for degradation. The same procedures were performed for L-PRF and T-PRF membranes without laser treatment.

**Result:**

Laser-treated L-PRF and T-PRF membranes showed lower degradation percentages compared to non-laser-treated membranes, but this difference did not reach statistical significance (*p* > 0.05). However, when laser treated L-PRF and T-PRF membranes were compared, the degradation percentage was significantly higher in L-PRF membrane (*p* < 0.05). Histologic examination showed that the fibrin network structure of the laser-applied L-PRF and T-PRF membrane groups was significantly denser than the non-laser-applied groups (*p* < 0.05). SEM analysis revealed that the fibrin network was denser, thicker and more complex in the laser-applied L-PRF and T-PRF membrane groups.

**Conclusion:**

In this study, the biostimulative effect of laser increased the fibrin network thickness, cross-link structure and density of L-PRF and T-PRF membranes. When the degradation percentages on the membranes were evaluated, no significant difference was observed between the groups.

**Clinical relevance:**

Understanding how laser photobiomodulation affects the structure and degradation resistance of both L-PRF and T-PRF membranes can guide clinicians in selecting the most suitable autologous biomaterial for enhancing wound healing and regenerative outcomes in dental procedures. The structure of PRF membranes used in dentistry can be improved using the biostimulative effect of the laser. This application may increase the use of these autologous and easily obtainable materials in treatments.

## Introduction

Photobiomodulation (PBM) refers to the use of low-level laser or light therapy to stimulate cellular functions through the activation of mitochondrial respiratory chain components and membrane photoreceptors [1, 2]. This interaction leads to increased production of adenosine triphosphate (ATP), modulation of reactive oxygen species (ROS), and initiation of signaling pathways that support tissue repair and regeneration [3, 4]. In dentistry, PBM has become an increasingly popular adjunctive tool, not only for its antimicrobial effects but also for its ability to enhance wound healing, reduce postoperative discomfort, and stimulate cell proliferation [3, 4].

The effectiveness of photobiomodulation (PBM) is closely associated with several key laser parameters, including wavelength, power output, energy density (fluence), irradiation duration, and beam profile [5, 6]. In support of this, Parker et al. emphasized the clinical importance of optimizing laser application parameters in their study, highlighting that proper adjustment is critical for therapeutic efficacy [5]. Diode lasers, particularly those emitting at 980 nm, are frequently used in dental PBM applications due to their favorable absorption characteristics and well-documented biostimulatory effects. However, studies have shown that other wavelengths, such as 635 nm, 405 nm, and 450 nm, can also enhance mitochondrial activity in fibroblasts, thereby demonstrating potential applicability in PBM [6]. The therapeutic effectiveness of diode lasers is quite wide, which increases the use of diode lasers in PBM applications. Although research has been conducted using a variety of wavelengths, the 980 nm wavelength stands out for its capacity to stimulate osteoblasts [7], promote fibroblast migration [8], and enhance collagen production [9]. Despite the lack of a universally accepted wavelength for PBM, lasers in the 800–980 nm range are generally preferred due to their deeper tissue penetration and consistent biological effects [10–12]. Since the choice of laser parameters directly influences outcomes, it is essential to select the most appropriate wavelength, duration, and power settings for effective PBM treatment.

Power output is another critical parameter. Output settings that are too low may be biologically ineffective, while excessive power can lead to cellular stress or thermal damage [12–14]. Amaroli et al. emphasized the importance of optimizing laser parameters to balance ROS modulation and mitochondrial activity, reporting that 0.2–0.5 W output supports biostimulation without cytotoxicity [9, 15]. In addition, Huang et al. and others have suggested that regenerative effects are most pronounced within the dose range of 5–25 J/cm², with a commonly accepted therapeutic window around 15 J/cm² [16–18]. To ensure optimal efficacy while minimizing tissue damage, the present study utilized a 980 nm diode laser at 0.5 W output and 15 J/cm² fluence, applied at a 1–2 mm working distance [19, 20].

Autologous platelet concentrates such as Platelet-Rich Fibrin (PRF) have emerged as valuable biomaterials in regenerative dentistry due to their inherent biological properties [21, 22]. PRF promotes tissue healing through a fibrin matrix enriched with leukocytes, platelets, and growth factors that are slowly released over time [23–30]. The classic form, known as Leukocyte- and Platelet-Rich Fibrin (L-PRF), is typically prepared using glass tubes and a standardized centrifugation protocol [24, 31]. However, concerns about silica contamination from glass tubes have led to the development of alternative methods [32]. One such advancement is Titanium-Prepared PRF (T-PRF), which replaces glass with titanium tubes during preparation, resulting in a more dense, homogeneous, and biocompatible fibrin architecture [33]. Comparative studies have shown that T-PRF may exhibit improved mechanical stability, slower degradation rates, and enhanced regenerative performance compared to L-PRF [34–36].

Despite widespread clinical use of both PRF variants, limited evidence exists regarding how they respond to external modulatory stimuli such as laser therapy. Understanding the interaction between PBM and PRF membranes could be crucial for optimizing their combined use in clinical applications. For instance, alterations in the fibrin network or degradation rate could significantly affect their biological performance.

Therefore, the present in vitro study aimed to investigate the effect of diode laser photobiomodulation on the morphological characteristics and degradation resistance of L-PRF and T-PRF membranes. By identifying whether PBM enhances or compromises the structural integrity of PRF membranes, this research intends to support clinicians in selecting appropriate regenerative protocols and improve the predictability of outcomes in dental treatments.

## Materials and methods

The study protocol was carried out according to the principles described in the Declaration of Helsinki, including all changes and revisions. Approval for this study was obtained from Bolu Abant Izzet Baysal University Clinical Research Ethics Committee (Decision No: 2023/231).

Considering the possible losses, the minimum sample size was determined as 15 for each group.

### Study design and experimental groups

The study was conducted on 15 healthy volunteers (both male and female) aged between 18 and 65 years. From each participant, a total of 8 blood samples were collected, resulting in a total of 120 blood samples used for analysis.

The samples were randomly divided into four experimental groups (*n* = 30 samples per group), as follows.

- 1. Group 1 – L-PRF without laser (Control Group A): 
  - 30 blood samples processed to obtain Leukocyte- and Platelet-Rich Fibrin (L-PRF) without the application of photobiomodulation (PBM).
- 2. Group 2 – L-PRF with laser (Test Group A):
  - 30 blood samples processed to obtain L-PRF, followed by the application of PBM using a diode laser.
- 3. Group 3 – T-PRF without laser (Control Group B):
  - 30 blood samples processed to obtain Titanium-Prepared Platelet-Rich Fibrin (T-PRF) without laser treatment.
- 4. Group 4 – T-PRF with laser (Test Group B):
  - 30 blood samples processed to obtain T-PRF, with subsequent laser irradiation applied.

Each group consisted of equal numbers of samples (*n* = 30), and all procedures were standardized to ensure consistent processing and irradiation conditions.

### Inclusion criteria

- Between the ages of 18–65.
- Systemically healthy.
- Not taking any medication on a regular basis.
- No previous history of smoking or alcohol.

### Exclusion criteria

- Smokers (tobacco) and alcohol users.
- Those under bisphosphonate use.
- Those with platelet function and coagulation disorders.
- People taking medication that affects the natural clotting process.
- Pregnant and lactating women.

The aim of this study was to measure the changes in the amount of degradation of T-PRF and L-PRF fibrin structures, which are widely used in dentistry, by utilizing the biostimulative efficiency of the laser, and to examine the histological and scanning electron microscope images.

### Platelet and complete blood count

Patients who agreed to participate in the study and met our inclusion criteria were referred to their family physicians for platelet and complete blood counts before any procedure was performed.

### Blood collection from individuals and obtaining PRFs

In our clinic, blood was collected from the antecubital vein into 4 empty vacuum tubes by experts using a vacutainer set by following the asepsis and antisepsis rules routinely observed. 2 weeks later, a second blood draw of 40 mL was performed from the right and left antecubital veins of the same patients using a 20 mL syringe, divided equally into 10 mL titanium tubes. This two-step approach was chosen to avoid the collection of large blood volumes (80 mL) in a single session.

**Fig. 1:**
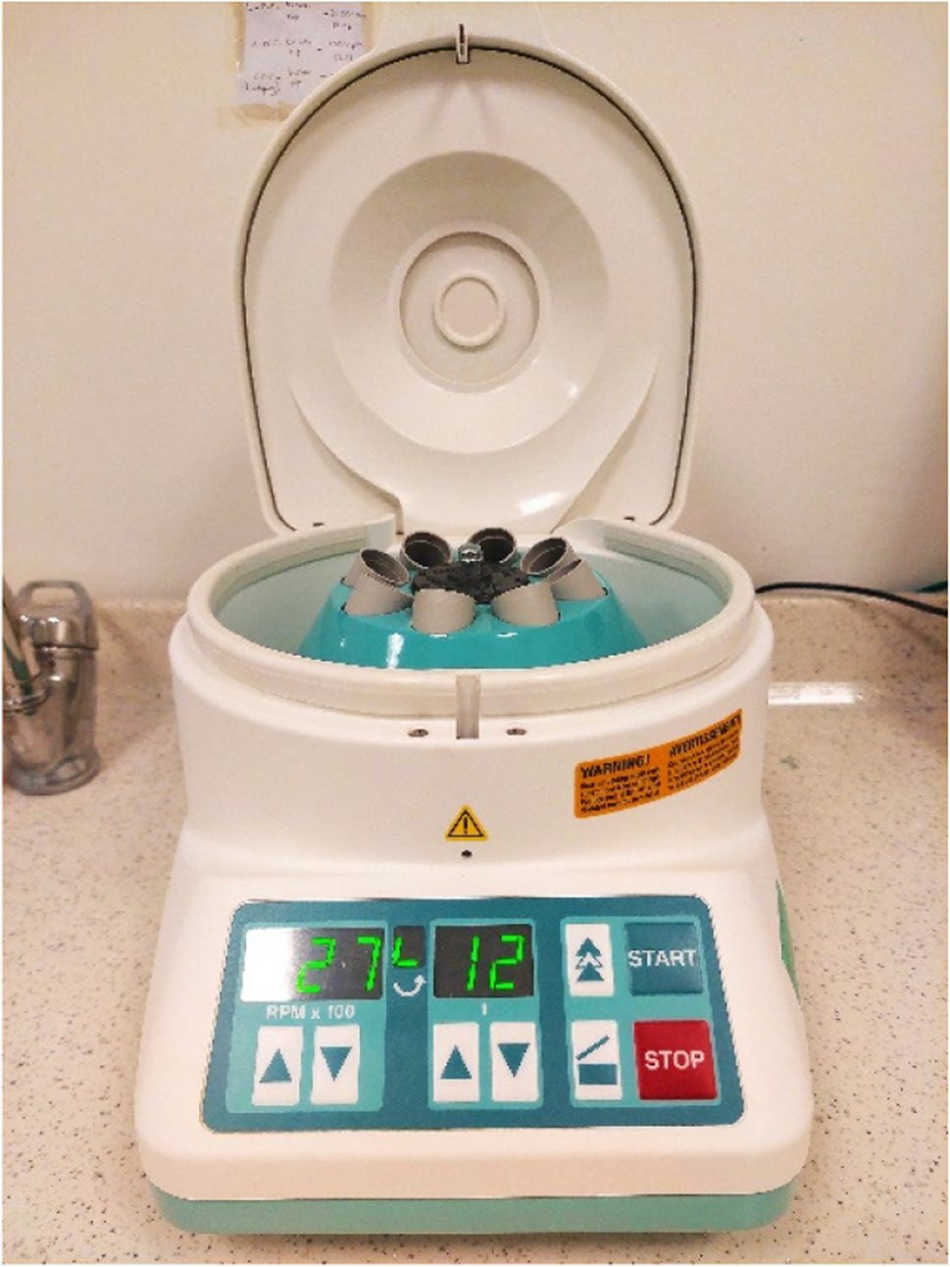
IntraSpinTM centrifuge

The blood collected from the individuals in 10 mL vacuum empty tubes was placed reciprocally in the IntraSpin™ (Intra-Lock International, USA) for centrifugation and the device was operated at 2700 rpm for 12 min (Fig. 1).

**Fig. 2:**
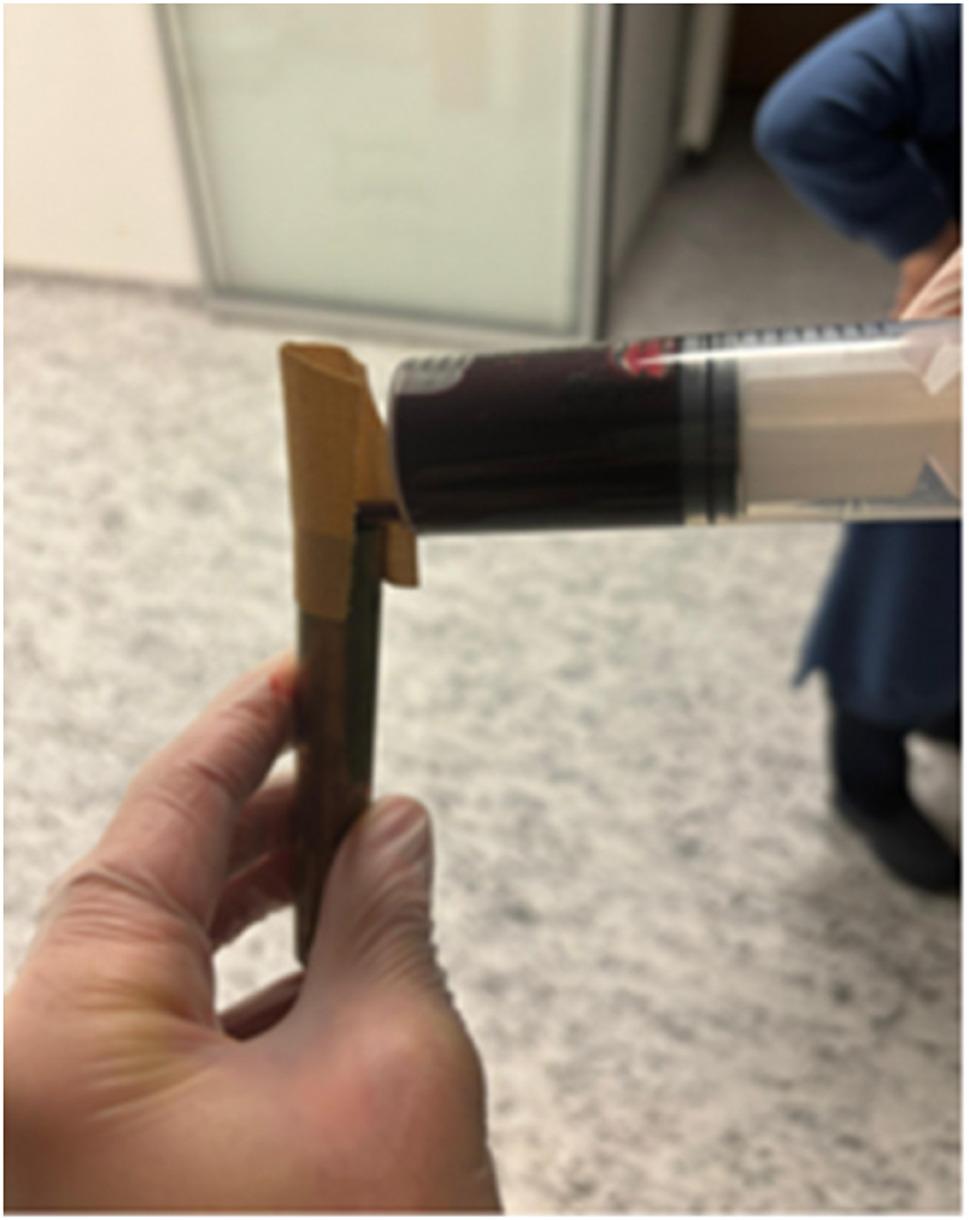
Obtaining T-PRF from individuals

The 40mL blood samples taken from the individuals 2 weeks later were divided into titanium tubes as 10 mL each and the device was operated at 2700 rpm and 12 min for centrifugation (Fig. 2).

The methods of obtaining L-PRF and T-PRF fibrins obtained in this study are summarized in Table 1.

**Table 1:** Centrifugation protocols for L-PRF and T-PRF fibrins

Parameter	L-PRF#	T-PRF*
Tube material	Vacumm empty plastic tubes	Grade IV titanium tubes
Centrifugation speed	~2700 rpm	~2700 rpm
Centrifugation time	12 minutes	12 minutes

(# Dohan Ehrenfest DM, Rasmusson L, Albrektsson T. Classification of platelet concentrates: from pure platelet-rich plasma (P-PRP) to leucocyte- and platelet-rich fibrin (L-PRF). Trends Biotechnol. 2009;27(3):158-67., *Tunalı M, Özdemir H, Küçükodacı Z, Akman S, Yaprak E, Toker H, et al. A novel platelet concentrate: titanium-prepared platelet-rich fibrin. Biomed Res Int. 2014;2014:209548)

**Fig. 3:**
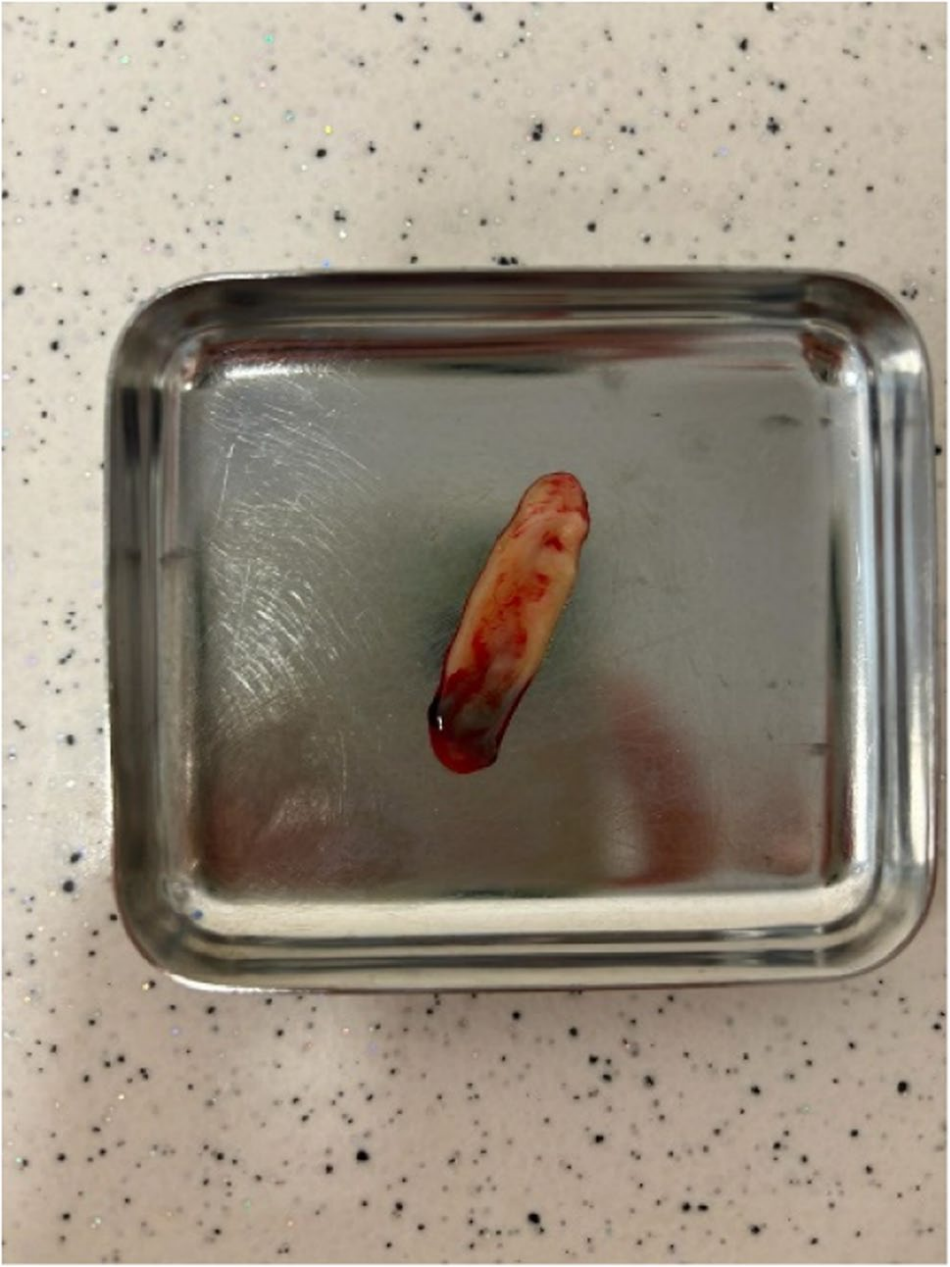
L-PRF membrane

After centrifugation, the fibrin structure in the middle of the 3 layers formed in the tubes was removed with a pressel. The fibrin layer was transferred to PRF box (Xpression™ Box Kit) to obtain membranes. Magnetic plates of 1 mm thickness were used to standardize the thickness of the membranes and the fibrin was kept in the PRF box for 2 min (Fig. 3).

### Modification of L-PRF and T-PRF Membranes by Photobiomodulation

**Fig. 4:**
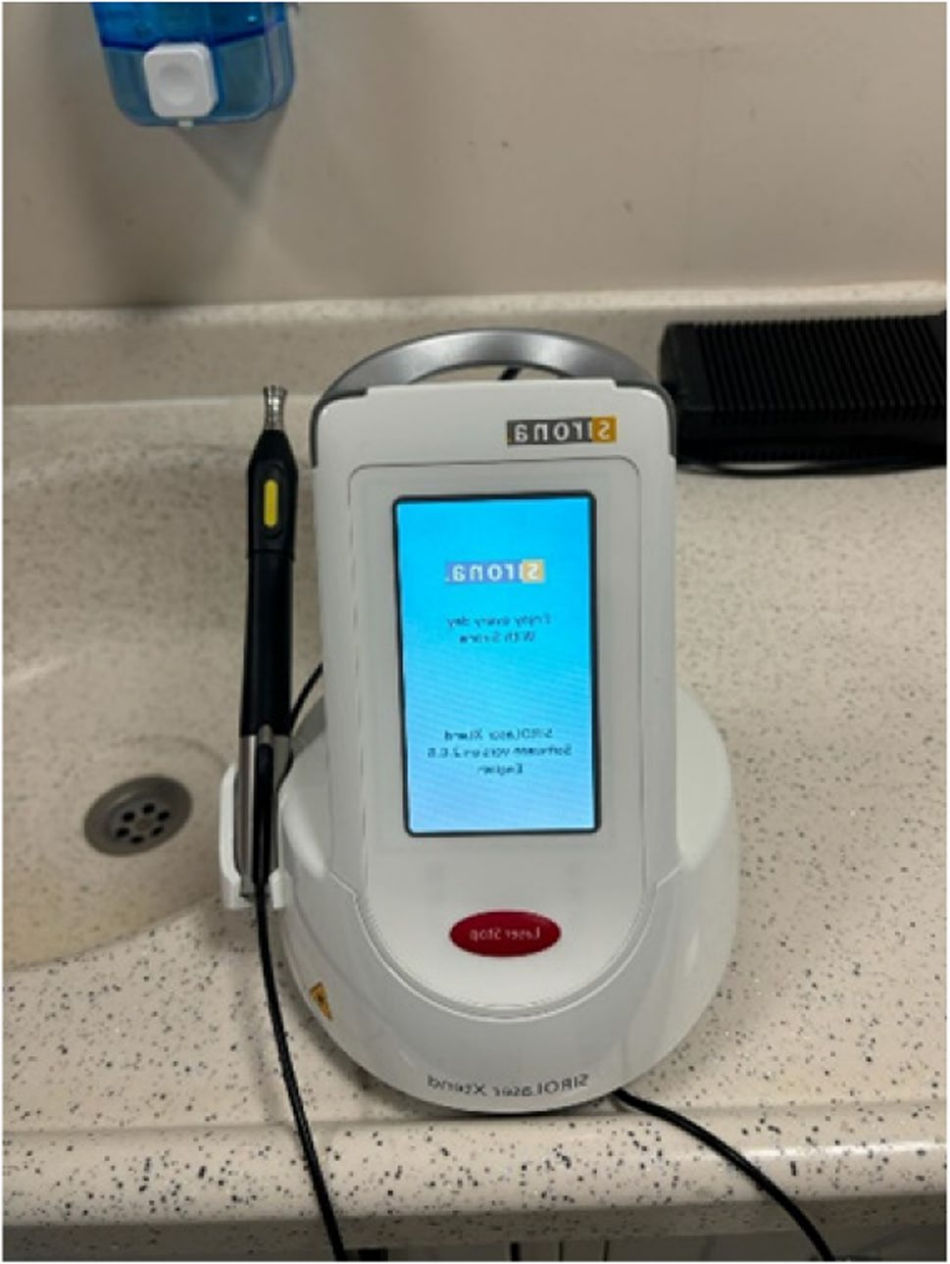
Sirona Laser Xtend device

**Fig. 5:**
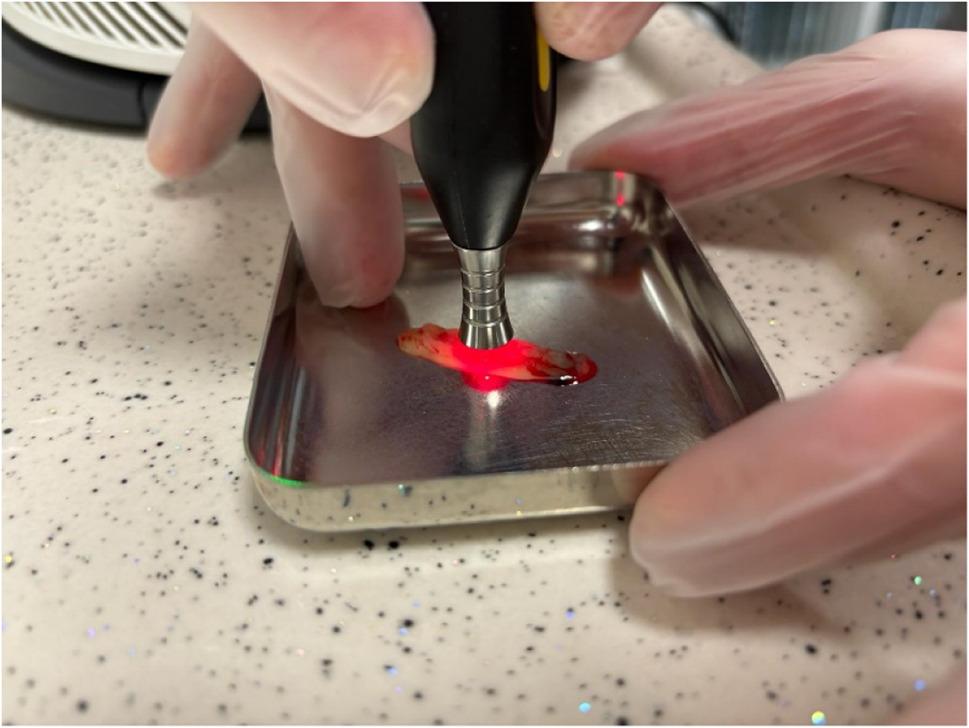
Laser application to PRF membrane at a distance of 1-2 mm

Two of the four L-PRF and T-PRF membranes obtained from each donor were subjected to photobiomodulation using a Sirona Laser Xtend diode laser operating at 980 nm in continuous wave mode. The irradiation was applied to a single surface of each membrane, with the laser positioned at a 90-degree vertical angle in point-mode (non-scanning) from a fixed non-contact distance of 1–2 mm for a total exposure duration of 3 min (Figs. 4 and 5).

During irradiation, samples were placed in a metallic tray to maintain stability and facilitate handling. Preliminary comparative testing of different tray materials, including glass and plastic, indicated that the metallic tray ensured the most consistent and effective laser energy distribution. A stationary, spot-irradiation technique was employed, with the laser beam directed perpendicularly and centrally onto each PRF membrane without any lateral movement or scanning. This approach ensured uniform and reproducible energy delivery across all irradiated samples.

All laser applications were conducted under identical ambient conditions and performed by the same trained operator to minimize procedural variability.

Although it is acknowledged that the reflective properties of the metallic tray might locally influence laser energy distribution and potentially cause minor temperature increases, this setup was selected based on empirical evidence from preliminary tests, which demonstrated optimal performance in terms of laser application efficiency and sample integrity.

Following irradiation, one of the PBM-treated L-PRF and T-PRF membranes was vertically bisected using sterile surgical scissors. One half was allocated for histological processing, and the other for scanning electron microscopy (SEM) analysis. The remaining PBM-treated membranes were used for degradation studies. The same procedures were identically applied to the non-irradiated L-PRF and T-PRF control membranes to ensure comparability.

### Histological Examination of L-PRF and T-PRF Membranes

L-PRF and T-PRF samples were prepared for histological analysis using the cell block cytology technique [39]. The isolated PRF membranes were carefully placed into small sterile gauze pouches, labeled with patient identifiers, and then positioned into standard tissue cassettes. These cassettes were immersed in 10% neutral buffered formalin solution at room temperature (approximately 22–24 °C) for 24 h to ensure optimal fixation and preservation of tissue morphology.

Following fixation, the tissue cassettes were transferred to a separate stainless-steel container and processed using an automated tissue processor (Leica ASP 300 S). The dehydration and clearing steps were performed in the following sequence:

- 10% formalin: 2 hours
- Alcoholic formalin: 1.5 hours
- Alcoholic formalin: 1 hour
- 95% ethanol: 1 hour (under vacuum)
- 95% ethanol: 45 minutes
- Absolute ethanol: 45 minutes (under vacuum)
- Absolute ethanol: 1 hour
- Xylene: 1 hour
- Xylene: 1 hour (under vacuum)

Subsequently, samples were embedded in paraffin using Leuckart molds, and 3 μm-thick tissue sections were obtained using a rotary microtome (Leica RM2255) for further histological staining and evaluation.

The deparaffinization step involved heating the slides to 55 °C, followed by immersion in xylene to remove the paraffin, preparing the sections for histological staining.

The sections were evaluated by a pathologist under various magnifications in a LEICA DM 2000 LED light microscope.

L-PRF and T-PRF membranes were microscopically analyzed using the blood element adhesion index (BEAI) [37].

- Score 0: Absence of fibrin mesh.
- Score 1: Rarely dispersed fibrin network.
- Score 2: Thin fibrin mesh that weakly interweaves with each other.
- Score 3: Dense network of densely interlaced fibrin.
- Score 4: Thick fibrin network with very dense interweaving.

Hematoxylin and eosin stained sections were photographed at different magnifications with the INFINITY 3 ANALYZE Release 6.5 imaging system.

### Scanning Electron Microscopy (SEM) analysis of L-PRF and T-PRF membranes

Scanning electron microscopy (Zeiss Gemini 500, Germany) was used for morphological evaluation of L-PRF and T-PRF membranes. L-PRF and T-PRF membranes were fixed with 2.5% glutaraldehyde and then dried. The samples were fixed on a slide surface to visualize the fibrin network structure in the samples. The membranes were coated with 20 nm gold/palladium sputtering before SEM examination and photographs were obtained and recorded under x2000, x5000 and x10000 magnifications. These magnification levels were chosen to enable evaluation of both general topographical features and finer ultrastructural details such as fibrin network organization and cellular components. A specialist in image interpretation quantified the images, taking into account parameters such as fibrin network density, cross-linking structures, and fibrin network thickness.

### Degradation of L-PRF and T-PRF membranes in PBS solution

The weights of L-PRF and T-PRF membranes were measured in grams on a precision balance (Sartorius CP225D Analytical Balances).

**Fig. 6:**
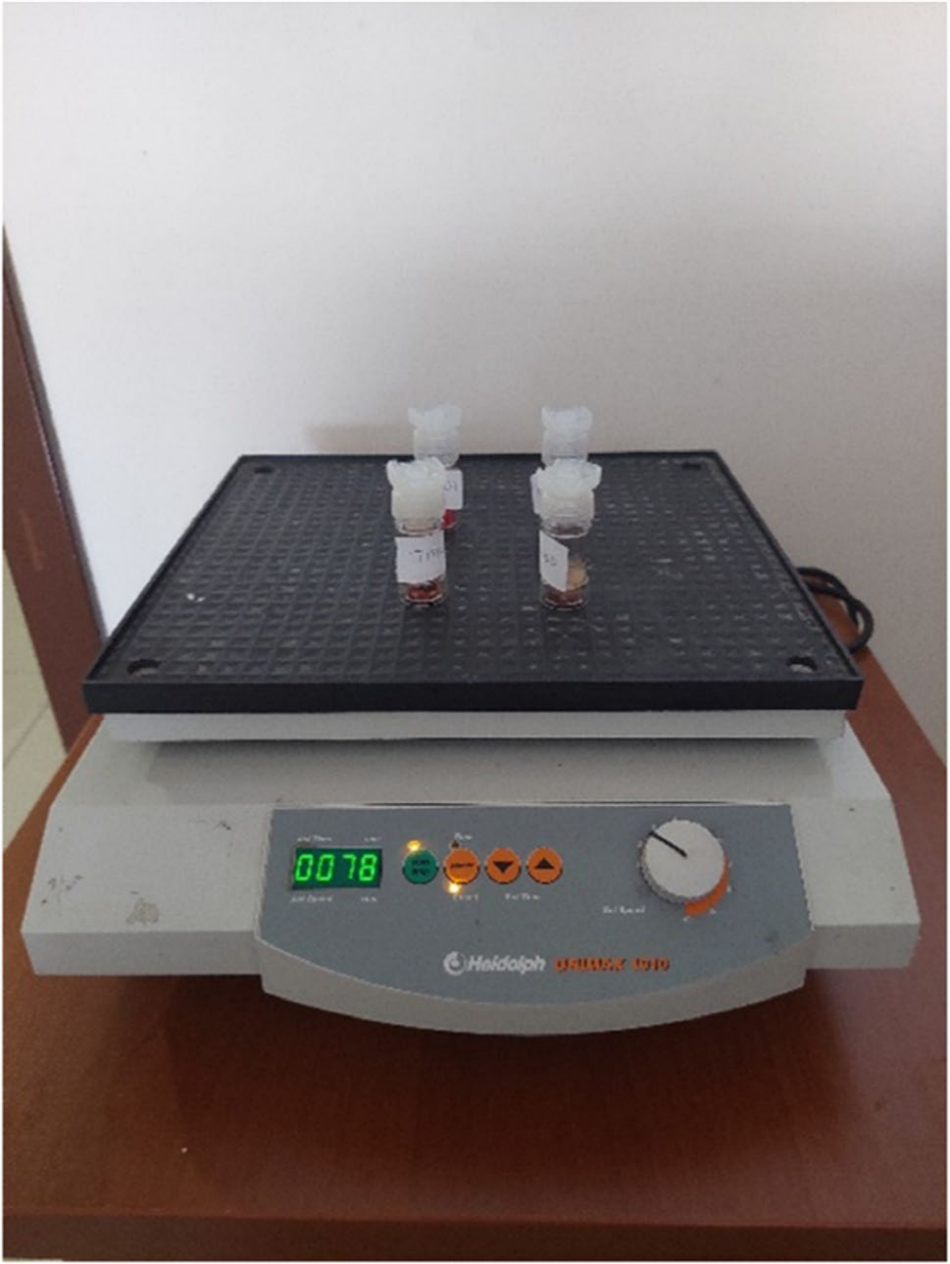
Degraded samples (Unimax 1010 Orbital Shaker)

L-PRF and T-PRF membranes were subjected to continuous shaking in 7.4% PBS solution for 1 week in an orbital shaker (Unimax 1010 Orbital Shaker) to evaluate the degradation rates (Fig. 6).

At the end of 1 week, the membranes were washed with distilled water, dried and weighed again in grams on a precision balance. The following formula was used to calculate the amount of degradation;

Amounts of degradation: % decay = $\:\frac{initial\:weight-final\:weight}{initial\:weight}X100$ [38].

### Statistical Analysis

Statistical analyses were performed with IBM SPSS 25 computer program. The normal distribution of the data was analyzed by Shapiro-Wilk test. Chi-Square test was used to analyze categorical variables. One-Way Anova test was used for normally distributed data between groups and Nonparametric Permanova test was used for non-normally distributed data. Values with *P* < 0.05 were considered statistically significant.

## Results

### Demographic Findings

This study was systemically performed on T-PRF and L-PRF membranes obtained from 15 healthy individuals. This study was planned as a total of 4 groups: laser treated L-PRF and T-PRF membranes and non-laser treated L-PRF and T-PRF membranes. The fibrin network structures, scanning electron microscope (SEM) images and the amount of degradation and chemical degradation of laser-treated and non-laser-treated L-PRF and T-PRF membranes were evaluated by light microscopy.

Fifteen patients aged between 18 and 65 years were included in this study. The number, age and gender distribution of the patients are shown in Table 2. The mean age of the study participants was 33.33 ± 12.01 years. No statistically significant difference was observed between the mean age of the men (34.37 ± 16.01) and women (32.14 ± 5.87) (*p* > 0.05).

**Table 2:** Demographic information of the individuals participating in the study

	Number	Age (Mean±Sd)	Median	Min.	Max.
Total	15	33,33±12,01	32	21	65
Male	8 (%53,3)	34,37±16,01	28	21	65
Female	7 (%46,7)	32,14±5,87	32	23	41
*P* value	0,602				

Data are expressed as mean ± standard deviation (SD). Mann-Whitney U Testi

### Individuals’ blood count values

Complete blood counts were performed in all patients who participated in this study. The mean blood parameters of the patients are shown in Table 3. All blood parameters of the patients were within the normal range.

**Table 3:** Blood parameters of individuals

	N	Mean±Sd	Median	Min.	Max.
WBC	15	6,74±1,87	6,71	5,00	8,81
RBC	15	4,93±0,42	4,93	4,20	5,71
HCT	15	41,66±3,57	42,20	32,40	47,80
PLT	15	274,86±58,62	276	171	405
MCV	15	84,66±7,12	85,80	63,90	93,10
PDW	15	13,87±2,76	14,80	9,70	17,20
MPV	15	9,96±1,19	9,60	8,20	12,30

Data are expressed as mean ± standard deviation (SD). (WBC: Leukocyte count, RBC: Erythrocyte count, *HCT* Hematocrit, *PLT* Platelet count, *MCV* Mean erythrocyte volume, *PDW* Platelet distribution width, *MPV* Mean platelet volume) descriptive statistics

### Chemical degradation analysis

The weights of L-PRF and T-PRF membranes obtained from the patients were calculated in grams before and after degradation. The amount of chemical degradation of the membranes was obtained as a percentage by subtracting the initial weight from the final weight and dividing this result by the initial weight.

The mean weights of the L-PRF and T-PRF membrane groups with and without laser treatment before and after degradation are shown in Table 4.

**Table 4:** Weight averages of L-PRF and T-PRF groups before and after degradation

	L-PRF-		L-PRF+		T-PRF-		T-PRF+	
	Mean ± Sd	Median	Mean ± Sd	Median	Mean ± Sd	Median	Mean ± Sd	Median
**Pre-degradation**	0,264 ± 0,057	0,241	0,256 ± 0,087	0,240	0,323 ± 0,156	0,378	0,326 ± 0,133	0,296
**Post-degradation**	0,176 ± 0,090	0,170	0,207 ± 0,083	0,185	0,279 ± 0,142	0,336	0,291 ± 0,120	0,267

*L-PRF-* L-PRF group without laser treatment, *L-PRF+* Laser-treated L-PRF group, *T-PRF-* T-PRF group without laser treatment, *T-PRF+* Laser treated T-PRF group, *Mean* Mean,* SD* Standard deviation (Wilcoxon test)

The mean and standard deviation values of chemical degradation of all groups are shown in Table 5.

**Table 5:** Mean percentage of chemical degradation of all groups

	N	Mean ± Sd	Min.	Max
**Degradation (%)**	60	20,046 ± 2,565	1,13	94,81

(descriptive statistics)

When the chemical degradation percentages of the groups were compared, it was seen that the laser treated groups were less chemically degraded. L-PRF membranes without laser application were significantly more degraded than T-PRF membranes without laser application (*p* < 0.05). However, laser-treated T-PRF membranes were statistically significantly less chemically degraded than PBM and non-PBM-treated L-PRF membranes (*p* < 0.05) (Table 6).

**Table 6:** Comparison of degradation percentages of L-PRF and T-PRF membranes with and without laser treatment

		Mean±Sd		*P* Value
L-PRF-	L-PRF+	34,808±7,117	19,740±3,667	0,099
L-PRF-	T-PRF-	34,808±7,117	16,203±4,337	0,007*
L-PRF-	T-PRF+	34,808±7,117	11,100±2,402	<0,001*
L-PRF+	T-PRF-	19,740±3,667	16,203±4,337	0,089
L-PRF+	T-PRF+	19,740±3,667	11,100±2,402	0,035*
T-PRF-	T-PRF+	16,203±4,337	11,100±2,402	0,367

*L-PRF-* L-PRF group without laser treatment, *L-PRF+* Laser-treated L-PRF group, *T-PRF-* T-PRF group without laser treatment, *T-PRF+* Laser-applied T-PRF group. *p<0,05 (Independent samples t-test)

### Results of Histological Examination under Light Microscope

L-PRF and T-PRF membranes were analyzed microscopically on hematoxylin-eosin stained slides using the blood cell adhesion index (BEAI). As a result of this analysis, the membranes were scored with numbers ranging from 0 to 4 according to their fibrin density. The mean fibrin network pattern scores of the groups are shown in Table 7.

**Table 7:** Mean fibrin network pattern score of the groups

	N	Mean±Sd	Min.	Max.
L-PRF - fibrin mesh pattern score	15	1,933±0,593	1	3
L-PRF + fibrin mesh pattern score	15	2,533±0,743	2	4
T-PRF - fibrin mesh pattern score	15	2,066±0,703	1	4
T-PRF + fibrin mesh pattern score	15	2,866±0,777	2	4

*L-PRF-* L-PRF group without laser treatment, *L-PRF+* Laser-treated L-PRF group, *T-PRF-* T-PRF group without laser treatment, *T-PRF+* Laser-applied T-PRF group (Wilcoxon test)

A statistically significant denser fibrin network was observed in laser-applied membranes (*p* < 0.05), whereas a more disorganized and thin fibrin network was observed in non-laser-applied membranes (Table 8).

**Table 8:** Fibrin mesh pattern score distribution of groups

		L-PRF-	L-PRF+	T-PRF-	T-PRF+	Total	*P* Value
**Fibrin mesh score** **1–2**	**Number**	13_a_	9_a, b_	13_a_	5_b_	40	**0**,04*
	**% distribution**	%86,7	%60,0	%86,7	%33,3	%66,7	
**Fibrin mesh score** **3–4**	**Number**	2_a_	6_a, b_	2_a_	10_b_	20	
	**% distribution**	%13,3	%40,0	%13,3	%66,7	%33,3	

*L-PRF-* L-PRF group without laser treatment, *L-PRF+* Laser-treated L-PRF group, *T-PRF-* T-PRF group without laser treatment, *T-PRF+* Laser-applied T-PRF group. *p<0,05 (Chi-square test)

### Scanning Electron Microscopy (SEM) Analysis

For the evaluation of SEM images, images were obtained using the IMAGE software and interpreted at ×2000, ×5000, and ×10,000 magnifications by an experienced examiner with prior expertise in this field.

**Fig. 7:**
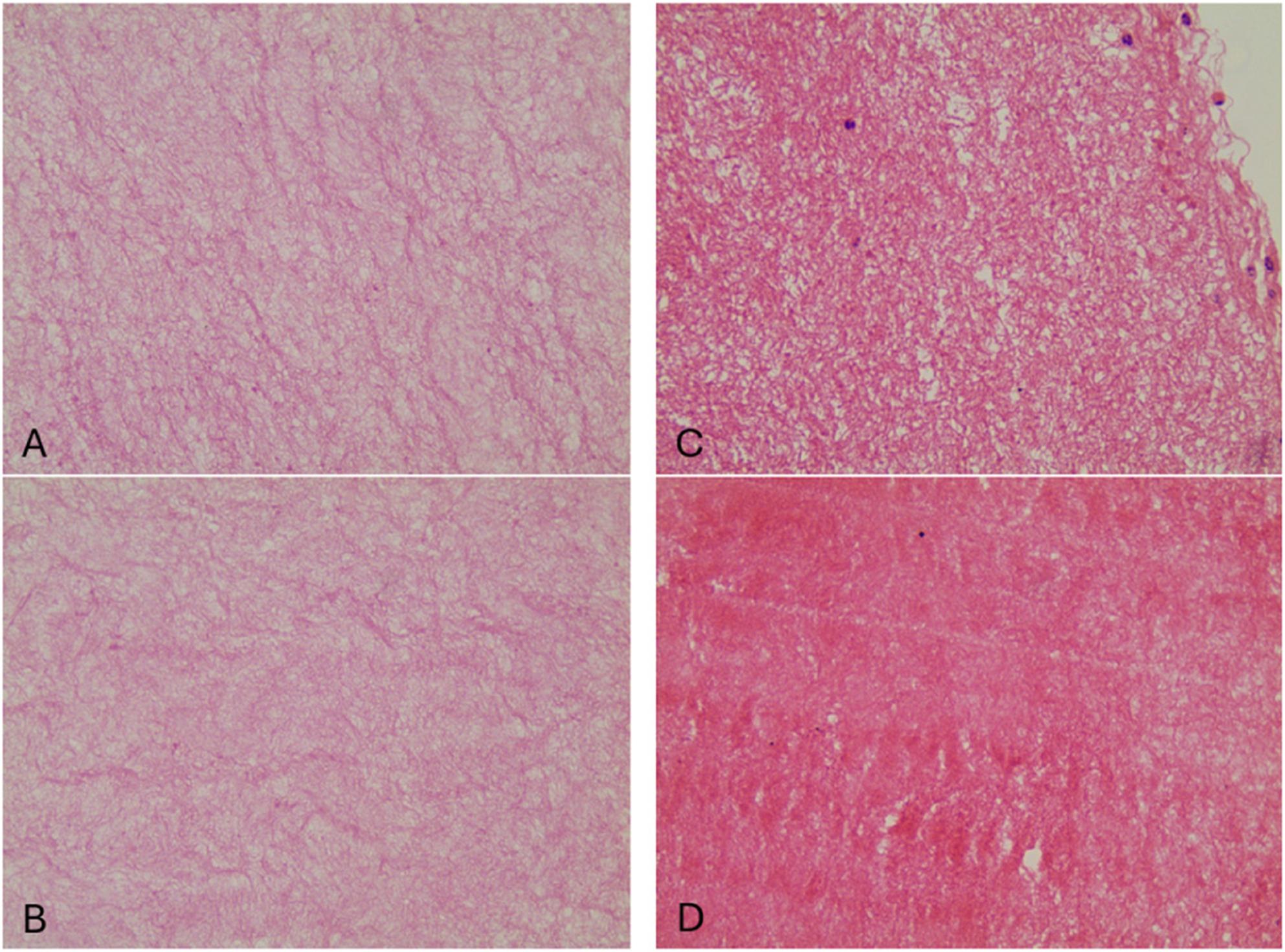
**A**: x400 magnification image of L-PRF membrane without laser application, **B**: x400 magnification image of L-PRF membrane with laser application, **C**: x400 magnification image of T-PRF membrane without laser application, **D**: Image at x400 magnification of laser applied T-PRF membrane

**Fig. 8:**
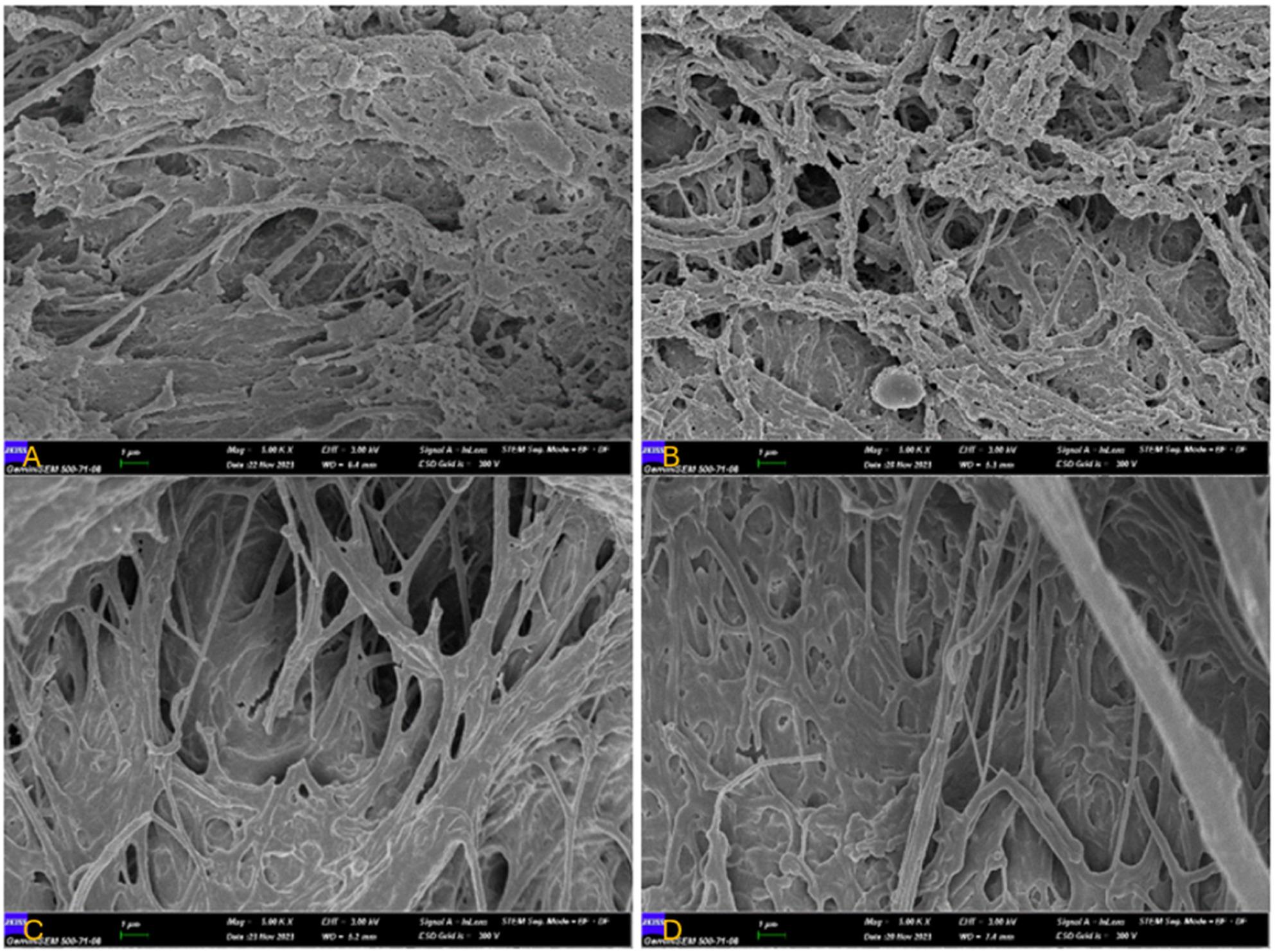
**A**: x5000 magnification image of L-PRF membrane without laser, **B**: x5000 magnification image of laser treated L-PRF membrane, **C**: x5000 magnification image of non-laser treated T-PRF membrane, **D**: x5000 magnification image of laser treated T-PRF membrane

As a result of SEM examination in this study, a mature fibrin network was observed in all groups. It was observed that the fibrin networks of T-PRF membranes were thicker and the fibrin network structure was more tightly textured compared to L-PRF membranes in all groups. PBM L-PRF and T-PRF membranes were observed to have more complex network structures compared to non-PBM L-PRF and T-PRF membranes Table 8, Fig. 7). Similarly, laser treated membranes were observed to have thicker primary and secondary fibrin strands than non-laser treated membranes (Fig. 8).

## Discussion

This in vitro study aimed to evaluate the biostimulative effects of diode laser irradiation on L-PRF and T-PRF membranes commonly used in dental practice. Although several studies have investigated the interaction between laser irradiation and platelet concentrates, to the best of this knowledge no previous work has comprehensively assessed all relevant parameters chemical degradation, fibrin network pattern, histological examination and SEM analysis characteristics in both L-PRF and T-PRF membranes simultaneously. Accordingly, the present study provides a more integrated analysis of the potential structural and degradative modifications induced by diode laser photobiomodulation in these two PRF variants.

The study population included systemically healthy male and female individuals aged between 18 and 65. Younger individuals were excluded due to ongoing growth and developmental changes that could affect blood parameters, while older individuals were excluded due to the higher likelihood of systemic disease and medication use, which could confound results.

L-PRF is widely used in dentistry due to its ease of production, high leukocyte content, and high antibacterial properties. In this study, L-PRF was preferred in this study because it has been studied extensively and is easy to obtain [39]. However, L-PRF is obtained by centrifuging blood in silica-containing plastic tubes. A study by Tsujinoe et al. demonstrated that silica particles in the tubes migrate into the membranes and may affect regenerative capacity [40]. Masuki et al. also reported that silica particles are cytotoxic to human periosteal cells and increase apitosis [41]. In light of these studies, T-PRF membranes, obtained using titanium tubes instead of silica tubes, were considered for more frequent use in dentistry as an alternative, and T-PRF membranes were also evaluated in add this study.

Laser biostimulation has been shown to enhance cellular activity, angiogenesis, and the synthesis of extracellular matrix components such as collagen. When applied in combination with PRF, a synergistic interaction occurs, enhancing wound healing through increased cellular-biomolecule interactions [42, 43].

In this study, PRF membranes were irradiated at a wavelength of 980 nm for 3 min. Since no similar study was found in the literature, a preliminary investigation into the duration was performed. Membranes were exposed to laser light for 1, 2, 3, and 5 min. Pathological examination revealed that the fibrin structure was affected by 3 min of irradiation, whereas no significant differences in fibrin networks were observed after other exposures. The 980 nm wavelength was chosen for biostimulation; although there are numerous studies on this topic [44–46], the reserchers no consensus has been reached.

Although no statistically significant difference in degradation percentages was observed among the four groups, a trend toward reduced degradation was evident with laser application. For example, degradation was 34.80% in the non-laser L-PRF group, while it decreased to 19.74% in the laser-treated L-PRF group. Similarly, T-PRF degradation decreased from 16.20% to 11.10% following laser application. These findings suggest that laser irradiation may slow fibrin degradation, although the lack of statistical significance may be attributed to the limited sample size. Notably, a significant difference was observed between the laser-treated L-PRF and T-PRF groups, with T-PRF exhibiting lower degradation. This result likely reflects T-PRF’s inherently slower resorption rate and denser fibrin structure, which may become further stabilized following photobiomodulation.

To date, no comparable study in the literature has examined the degradation profiles of laser-irradiated L-PRF and T-PRF membranes. However, supporting evidence exists from related investigations. Ravi et al. compared A-PRF, L-PRF, and T-PRF membranes, reporting the highest degradation in L-PRF (85%), followed by A-PRF (84%), and the lowest in T-PRF (82%) after 7 days of incubation in PBS. This study found that T-PRF was superior to A-PRF and L-PRF, although there was no significant difference in degradation percentages, similar to this study [38]. Saeed and colleagues found that the degradation rates in the 25 kGy gamma-irradiated and unsterilized fibrin membrane groups were higher than in the UV-sterilized and autoclaved membranes. This result highlights the role of external stimuli in structural stability [44]. Similarly, in a study comparing the degradation percentages of collagen membranes and PRF, PRF membranes showed a 36% weight loss after one week in PBS [45]. In their study, Isobe et al. found that PPTF exhibited a weaker cross-linking density in SEM images and underwent the most degradation [47]. In another study by Dereli Can et al., it was shown that amniotic membranes were degraded by approximately 85% in one week, while L-PRF and A-PRF lost approximately 31% and 40% of their initial weight, respectively [48]. Based on these findings, it can be inferred that the fibrin density and cross-linking structures of PRF membranes may be enhanced with PBM, leading to a slower resorption rate of the increased fibrin.

Recent findings underscore the differential but complementary effects of red (e.g., ~ 635 nm) and near‑infrared (NIR; e.g., ~ 808 nm) light in photobiomodulation. Kocherova et al. demonstrated that both wavelengths significantly improved viability and proliferation of human gingival fibroblasts, though the timing and magnitude of effects varied. Red light (~ 635 nm) was particularly effective after the first treatment, while repeated NIR exposure (~ 808 nm) more robustly influenced mesenchymal markers CD90 and CD105 in later sessions [49]. Based on these results, we believe that PBM may strengthen and increase secondary fibrin structures within fibrin, thereby reducing degradation rates. PBM can provide PRF membranes with a more durable structure and longer resorption times. Several studies have demonstrated that red light exposure (e.g., He-Ne laser at 632.8 nm or LEDs) prior to platelet activation can modulate the coagulation cascade by inhibiting aggregation induced by agonists such as ADP, collagen, epinephrine, PAF, fibrinogen, ristocetin, and TRAP [50, 51]. This suggests that PBM applied before centrifugation may alter platelet function and consequently influence the biological structure of the resulting.

Given the current limitations regarding parameter standardization (e.g., wavelength, fluence, duration) and the potential for systemic effects, this study employed PBM after PRF preparation, ensuring a controlled and reproducible application.

Histological evaluation in this study demonstrated that laser-treated membranes had significantly higher fibrin network pattern scores than non-laser groups. Notably, the score was higher in laser-treated T-PRF membranes than in their non-laser counterparts, supporting the structural reinforcement hypothesis. Similar findings have been associated with other biological factors. For instance, centrifugation protocols and donor age have been shown to influence fibrin density. A study by Mamajiwala et al. found that younger individuals (aged 20–34) produced PRF with denser fibrin networks, and higher centrifugation forces also led to denser fibrin [52]. Yajamanya et al. confirmed that aging results in looser fibrin structures, potentially due to decreased cellular components [53]. Chatterjee et al. examined PRF membranes from healthy individuals, smokers, and hypertensives, and found that systemic conditions contributed to a looser fibrin network in both L-PRF and T-PRF, though T-PRF retained a denser structure overall [54].

SEM imaging, offering a resolution up to 0.2 nm [55], provided detailed structural insights [56]. Laser-treated membranes displayed a denser and more complex fibrin network with thicker primary and secondary strands. These findings align with previous SEM-based studies. Gupta et al. observed that periodontitis and diabetes led to thinner, less organized fibrin networks in PRF clots [57]. Malgikar et al. reported that T-PRF had a significantly thicker and more organized fibrin matrix than L-PRF, attributing the difference to titanium’s superior biocompatibility [58].

Centrifugation settings are also known to influence fibrin architecture. In a study comparing various speeds and durations, PRF clots generated at higher centrifugal forces demonstrated denser fibrin matrices but lower cell concentrations [59]. Kobayashi et al. showed that compression technique also affects network density: PRF membranes compressed with gauze had denser fibrin networks compared to those processed with standardized devices [60]. Furthermore, horizontal versus fixed-angle centrifugation produced different PRF morphologies; fixed-angle techniques yielded PRFs with dense fibrin and red blood cell entrapment, while horizontal techniques resulted in networks enmeshing leukocytes and platelets [61].

Although recent studies have investigated the individual and combined effects of photobiomodulation (PBM) and platelet-rich fibrin (PRF) in tissue regeneration, there are also studies specifically focusing on the impact of PBM on human gingival fibroblasts. For instance, Bikmulina et al. (2020), in an in vitro study on mesenchymal cells derived from human gingiva, demonstrated that PBM at 840 nm significantly enhanced cell viability when applied within hydrogel matrices [62]. Similarly, another in vitro study examining various laser modalities reported increased proliferation and differentiation in human gingival fibroblasts following laser irradiation [5]. A study investigating the in vivo and in vitro effects of low-level laser therapy observed that it increased the proliferation rate and cellular migration of in vitro cultured human gingival fibroblasts. Conversely, laser application was reported to enhance cellular proliferation and attachment of osteoblast-like cells [63]. These findings provide additional support for the rationale behind add PBM application to enhance the regenerative capacity of PRF. The regenerative-promoting effects observed in vitro suggest that the efficacy of platelet concentrates—widely used in dental and periodontal treatments—may be further improved through adjunctive PBM.

While this study was conducted under in vitro conditions, it addresses a notable gap in the current literature and may serve as a foundation for future research exploring the biological interactions between PBM and PRF, particularly in more clinically relevant in vivo models.

It is important to acknowledge certain limitations when considering the generalizability of these results. Being an in vitro design, the results may not fully reflect in vivo conditions where biological variability and complex tissue interactions exist. There are studies showing decreases in fibrin density with age. The selection of a broad age group between 18 and 65 in othis study may have influenced fibrin network densities. The relatively small sample size may have limited statistical significance in some comparisons. Additionally, only one laser setting was tested, which may not represent all clinical scenarios. Further studies with larger samples, different parameters, and in vivo models are needed for broader conclusions.

One notable limitation of this study is the absence of precise temperature measurements during laser irradiation. Although a preliminary trial was conducted using both metal and glass containers, and no tactile temperature difference or histological alterations in PRF samples were observed, the lack of quantitative thermal data limits this ability to fully exclude the potential influence of heat conduction from the metallic tray. While any such effect is presumed to be minimal, it may have introduced a minor confounding factor in the experimental setup. In future studies on this topic, the heat setting should be precisely measured.

## Conclusions

In this study, the degradation percentages of PBM T-PRF and L-PRF membranes were found to be lower. It was shown in this study that the clinical resorption time of membranes can be prolonged by laser application.

In addition, it was shown that the thickness and tightness of the fibrin network structure increased in laser treated membranes, thus forming more complex structures. It shows that laser treatment can improve the biological and mechanical properties of PRF membranes, creating more durable and effective biomaterials for potential clinical applications. However, there are many factors affecting the fibrin structure. Therefore, many new studies are needed.

## Data Availability

Not applicable.

## Abbreviations

L-PRF: Leukocyte- and Platelet-Rich Fibrin
T-PRF: Titanium-Prepared Platelet-Rich Fibrin
PRF: Platelet Rich Fibrin
SEM: Scanning Electron Microscopy
PBM: Photobiomodulation
PBS: Phosphate Buffered Saline
BEAI: Blood Element Adhesion Index
nm: Nanometer
µm: Micrometer
J/cm^2^: Joules Per Square Centimeter
RPM: Revolutions Per Minute
W: Watt
mW: Miliwatt
InGaAs: Indium Gallium Arsenide
